# Effects of fish oil supplement on psoriasis: a meta-analysis of randomized controlled trials

**DOI:** 10.1186/s12906-019-2777-0

**Published:** 2019-12-05

**Authors:** Shih-Jyun Yang, Ching-Chi Chi

**Affiliations:** 1 Department of Dermatology, Chang Gung Memorial Hospital, Keelung, Taiwan; 2 Department of Dermatology, Chang Gung Memorial Hospital, Linkou, 5, Fuxing St, Guishan Dist, Taoyuan, 33305 Taiwan; 3 College of Medicine, Chang Gung University, Taoyuan, Taiwan

**Keywords:** Fish oil, Meta-analysis, Polyunsaturated fatty acids, Psoriasis, Systematic review

## Abstract

**Background:**

Fish oils, which contain omega-3 polyunsaturated fatty acids as the active ingredients, possess anti-inflammatory activities and may have therapeutic potential in diseases with an inflammatory etiology. Fish oil supplement has been advocated for treating psoriasis which is a chronic inflammatory dermatosis.

**Objective:**

We aimed to investigate the effects of fish oil supplement on psoriasis.

**Methods:**

We searched CENTRAL, Embase and MEDLINE on 24 January 2018 for randomized control trials (RCTs) on the effects of fish oil supplement in treating psoriasis. The Cochrane Collaboration’s tool was used to assess the risk of bias of included RCTs. We performed a random-effects model meta-analysis to obtain the pooled treatment effect estimates.

**Results:**

We included 13 RCTs with 625 participants. Three RCTs involving 337 participants provided usable data for meta-analysis. Fish oil supplement did not significantly reduce the severity of psoriasis when assessed by Psoriasis Area and Severity Index score (mean difference − 0.28; 95% confidence interval − 1.74 to 1.19).

**Conclusion:**

The current evidence does not support the use of fish oil supplement in treating psoriasis.

## Background

Psoriasis is a chronic inflammatory dermatosis characterized by well-demarcated erythematous plaques with silvery scales [1, 2]. Although the hallmark clinical feature is the cutaneous manifestation, psoriasis has increasingly been recognized as a systemic inflammatory disorder with comorbidities including arthritis [3], cardiometabolic disease [4], uveitis [5], and chronic kidney disease [6]. Psoriasis has substantial negative impact on affected patient’s quality of life [7].

One question frequently asked by psoriasis patients is whether a dietary change or supplementation with specific nutrients can improve their condition. Some studies have suggested that supplementation with fish oil, which contains omega-3 polyunsaturated fatty acids (ω-3 PUFAs) including eicosapentaenoic acid (EPA) and docosahexaenoic acid (DHA) as the active ingredients, may be beneficial in psoriasis, likely through their anti-inflammatory effect [8]. However, other studies have revealed conflicting results [9]. In this study, we aimed to systemically assess the evidence on the effects of fish oil supplement in treating psoriasis.

## Methods

We conducted a systematic review and meta-analysis of randomized controlled trials (RCTs) on the effects of fish oil supplement in treating psoriasis. The reporting of this study followed the Preferred Reporting Items for Systematic Reviews and Meta-Analyses (PRISMA) [10]. We searched the Cochrane Central Register of Controlled Trials (CENTRAL), Embase, and MEDLINE for relevant publications on 24 January 2018. The search strategy is listed in Table 1. Studies were included if they met all of the following eligibility criteria: (1) study design being RCTs; (2) the participants were psoriasis patients; (3) the study intervention was fish oil/ω-3 PUFAs supplement and the comparator was placebo or other active treatments; and (4) published in English. Studies involving only dietary modification were excluded. Our primary outcomes included: (1) the severity of psoriasis measured by Psoriasis Area and Severity Index (PASI) score or involved body surface area (BSA) and (2) adverse events (AEs). Our secondary outcomes included: (1) the degree of psoriasis signs including erythema (redness), scaling (desquamation), and induration (thickness/infiltration) and (2) the degree of pruritus.

**Table 1:** Search strategy

Database	Search strategy
Cochrane Central Register of Controlled Trials (CENTRAL)	#1 MeSH descriptor: [Psoriasis] explode all trees #2 Psoriasis:ti,ab,kw (Word variations have been searched) #3 #1 or #2 #4 MeSH descriptor: [Fish Oils] explode all trees #5 Fish Oil:ti,ab,kw (Word variations have been searched) #6 fish liver oil:ti,ab,kw (Word variations have been searched) #7 MeSH descriptor: [Cod Liver Oil] explode all trees #8 Cod Liver Oil:ti,ab,kw (Word variations have been searched) #9 MeSH descriptor: [Fatty Acids, Omega-3] explode all trees #10 Omega-3:ti,ab,kw (Word variations have been searched) #11 Omega3:ti,ab,kw (Word variations have been searched) #12 MeSH descriptor: [Eicosapentaenoic Acid] explode all trees #13 EPA:ti,ab,kw (Word variations have been searched) #14 eicosapentaenoic acid:ti,ab,kw (Word variations have been searched) #15 eicosapentaenoate:ti,ab,kw (Word variations have been searched) #16 icosapentaenoic acid:ti,ab,kw (Word variations have been searched) #17 MeSH descriptor: [Docosahexaenoic Acids] explode all trees #18 DHA:ti,ab,kw (Word variations have been searched) #19 docosahexaenoic acid:ti,ab,kw (Word variations have been searched) #20 docosahexaenoate:ti,ab,kw (Word variations have been searched) #21 #4 or #5 or #6 or #7 or #8 or #9 or #10 or #11 or #12 or #13 or #14 or #15 or #16 or #17 or #18 or #19 or #20 #22 #3 and #21
Embase	#1 ‘psoriasis’/exp. OR ‘psoriasis’ #2 ‘fish oil’ #3 ‘fish liver oils’ #4 ‘cod liver oil’ #5 ‘omega 3 fatty acid’ #6 ‘eicosapentaenoic acid’ #7 ‘icosapentaenoic acid’ #8 eicosapentaenoate #9 ‘docosahexaenoic acid’ #10 docosahexaenoate #11 #2 OR #3 OR #4 OR #5 OR #6 OR #7 OR #8 OR #9 OR #10 #12 #1 AND #11
MEDLINE	1 exp Psoriasis/ 2 psoriasis.mp. 3 1 or 2 4 exp. Fish Oils/ 5 fish oil.mp. 6 fish liver oil.mp. 7 exp. Cod Liver Oil/ 8 cod liver oil.mp. 9 exp. Fatty Acids, Omega-3/ 10 omega-3.mp. 11 omega3.mp. 12 exp. Eicosapentaenoic Acid/ 13 EPA.mp. 14 eicosapentaenoic acid.mp. 15 eicosapentaenoate.mp. 16 icosapentaenoic acid.mp. 17 exp. Docosahexaenoic Acids/ 18 DHA.mp. 19 docosahexaenoic acid.mp. 20 docosahexaenoate.mp. 21 or/4–20 22 3 and 21

Two authors (SY and CC) independently screened the titles and abstracts of search results to identify potentially eligible trials, and full texts of these studies were checked to determine whether they met our inclusion criteria. One author (SY) extracted the data from the included trials. If the data were incomplete in the text but may be extrapolated from the figure, we extracted them from the figure by using the WebPlotDigitizer Version 4.1 (Austin: Ankit Rohatgi, 2018). In studies which did not report the standard deviations for changes from baseline in continuous variables, we calculated a correlation coefficient from a study with detailed information and used it to impute the standard deviations using the following equation:

$S{D}_{change}=\sqrt{{\left(S{D}_{Baseline}\right)}^2+{\left(S{D}_{Endpoint}\right)}^2-2\times r\times S{D}_{Baseline}\times S{D}_{Endpoint}}$ , where *r* represents the correlation coefficient [11]. Another author (CC) verified these data.

One author (SY) assessed the risk of bias of included studies by using the Cochrane Collaboration’s tool [11] and the other author (CC) confirmed the judgment. The following items were categorized as having high, low or unclear risk of bias: random sequence generation, allocation concealment, blinding of participants and personnel, blinding of outcome assessment, incomplete outcome data, selective reporting, and other biases which focusing on baseline imbalance [11]. As to reporting bias, if a RCT did not report data on AEs, we rated it at high risk of selective reporting bias.

We used the Review Manager Version 5.3 (Copenhagen: The Nordic Cochrane Centre, The Cochrane Collaboration, 2014) in conducting meta-analysis. The random-effects model was employed due to anticipated clinical heterogeneity. Continuous outcomes were expressed as mean difference (MD) or as standardized mean difference (SMD) if different scales had been used to measure the same outcome. The statistical heterogeneity was assessed by calculating the I^2^ statistic.

## Results

### Characteristics of included studies

**Fig. 1:**
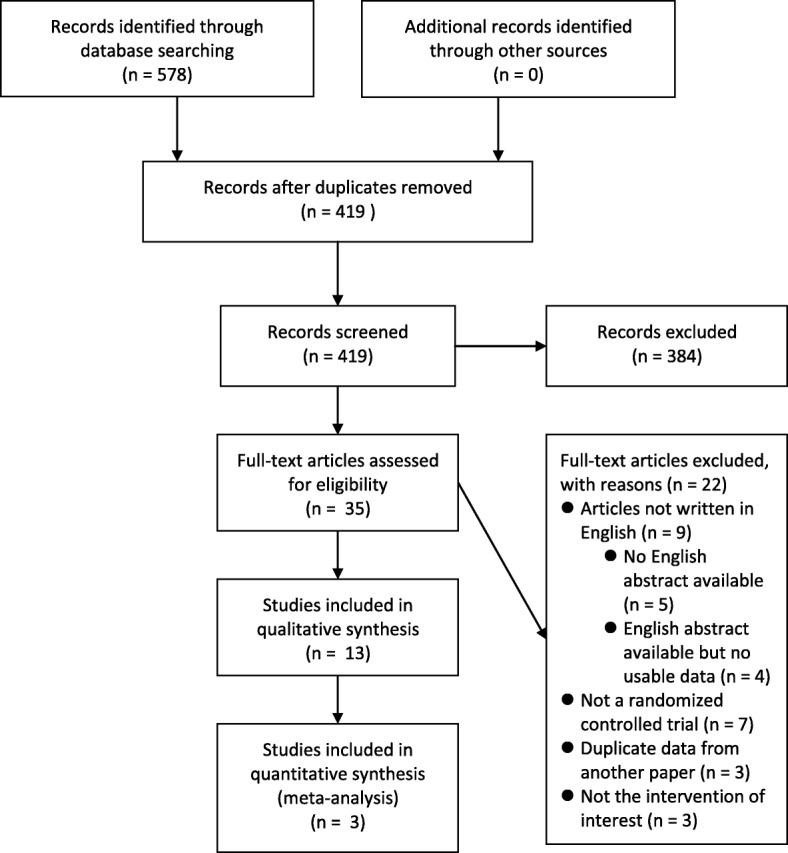
PRISMA study flow chart

The PRISMA study flow chart is illustrated in Fig. 1. Our search identified 419 articles after removing duplicates. Among them, 13 RCTs with 625 participants met our inclusion criteria and were included in this study [12–24]. The fish oil supplement was administered orally in the form of capsule or oil in 11 RCTs [12–14, 16–19, 21–24] and given intravenously as lipid emulsion in 2 RCTs [15, 20]. Three RCTs used capsules containing a combination of fish oil and evening primrose oil [21, 23, 24]. Five of the 13 included RCTs reported benefits of fish oil supplement in treating psoriasis [12, 14, 15, 17, 20]; the other eight RCTs, however, suggested fish oil supplement was not better than control treatment [13, 16, 18, 19, 21–24]. The characteristics of included RCTs are shown in Table 2. Three studies involving 337 participants provided usable data for meta-analysis [18, 20, 22].

**Table 2:** Characteristics of the included studies

Study ID	Participants	Intervention	Control	Outcome of interest
Bittiner 1988 [12]	Chronic stable plaque psoriasis.	MaxEPA capsules 10# daily (containing 1.8 g EPA) for 12 weeks.	Capsules containing olive oil 10# daily for 12 weeks.	Erythema, scaling, BSA, itching.
Bjørneboe 1988 [13]	Stable psoriasis vulgaris.	MaxEpa® capsules 10# daily (containing 1.8 g EPA and 1.2 g DHA) for 8 weeks.	Capsules containing olive oil 10# daily for 8 weeks.	Erythema, induration, desquamation, BSA, blood tests.
Danno 1998 [14]	Moderately-involved, chronic plaque-type psoriasis vulgaris.	20 mg etretinate combined with 1.8 g EPA /day for 12 weeks.	20 mg etretinate for 12 weeks.	Clinical score (based on erythema, induration, and scaling of 3 selected plaque lesions), AEs, routine lab data.
Grimminger 1993 [15]	Acute guttate psoriasis with BSA at least 10%.	50 ml fish oil derived lipid emulsion twice daily via IV route for 10 days (containing 2.1 g EPA + 2.2 g DHA per day).	50 ml soya oil derived lipid emulsion twice daily via IV route for 10 days.	Erythema, infiltration, desquamation, subjective score (based on appearance of lesions, impairment of daily life, pruritus, burn and pain), AEs, blood tests.
Gupta 1989 [17]	Stable plaque psoriasis with total BSA 10–50%; skin type II or III.	Max-EPA^R^ capsules 10# twice daily (daily total of 3.6 g EPA and 2.4 g DHA) for 15 weeks and UVB therapy for 8 weeks (weeks 3–11).	Olive oil capsules 10# twice daily for 15 weeks and UVB therapy for 8 weeks (weeks 3–11).	Erythema, thickness, scale, BSA, AEs.
Gupta 1990 [16]	Stable plaque type psoriasis with BSA at least 10%.	Topical betamethasone diproprionate twice day (45 g/week) + Max-EPA^R^ capsules 10# thrice daily (daily total of 5.4 g EPA and 3.6 g DHA) for 3 weeks then keep Max-EPA^R^ capsules.	Topical betamethasone diproprionate twice day (45 g/week) + olive oil capsules 10# thrice daily for 3 weeks then keep olive oil capsules.	Global severity score (based on scale, erythema and thickness), BSA, AEs.
Kristensen 2018 [18]	Psoriatic arthritis; adult above 18 years of age.	Daily intake of 6 capsules containing 3 g of n-3 PUFA (50% EPA + 50% DHA) for 24 weeks (1.5 g EPA + 1.5 g DHA per day).	Daily intake of 6 capsules containing 3 g of olive oil for 24 weeks.	VAS-pain, Health Assessment Questionnaire, Disease Activity Score based on CRP, tender joint count (68), swollen joint count (66), ASDAS, BASDAI, BASMI, LEI, SPARCC, PASI, NSAID (no. of tablets/week), Paracetamol (no. of tablets/week), lab tests, AEs.
Madland 2006 [19]	Polyarticular psoriatic arthritis.	10 ml seal oil self administered orally before meals thrice daily for 14 days (2.4 g EPA, 1.1 g DPA, 2.6 g DHA per day).	10 ml soy oil self administered orally before meals thrice daily for 14 days.	Joint pain intensity, patient’s global assessment, number of tender and swollen joints, PASI, lab tests.
Mayser 1998 [20]	Inpatients between 18 and 80 years of age with chronic plaque psoriasis; PASI ≥15.	Infusions with a ω-3 fatty acid–based lipid emulsion (Omegavenous) 100 ml twice daily for 14 days (4.2 g of both EPA and DHA/day).	Infusions with a conventional ω-6-lipid emulsion (Lipovenous) 100 ml twice daily for 14 days.	PASI, PASI 50, intensity of psoriasis, self assessment (VAS), AEs, SAEs, lab tests.
Oliwiecki 1994 [21]	Chronic stable plaque psoriasis; 16–70 years old.	Placebo capsules (500 mg liquid paraffin) 6# bid for 4 weeks then receiving active treatment (capsules containing 430 mg evening primrose oil + 107 mg fish oil + 10 mg vitamin E; 6# bid) for the next 24 weeks (1.284 g fish oil per day).	Placebo capsules (500 mg liquid paraffin) 6# bid for 28 weeks.	10 cm linear analogue scale to measure erythema, scaling and overall severity; plaque thickness; patient self-assessment.
Søyland 1993 [22]	Stable plaque psoriasis with BSA > 8%.	6 capsules daily, each containing 1 g of highly concentrated ethyl esters of very-long-chain n-3 fatty acids (3.06 g EPA + 1.92 g DHA per day), for 4 months.	6 capsules of corn oil daily, each containing 1 g, for 4 months.	PASI, erythema, infiltration, desquamation, subjective score based on degree of redness, scaling, itching and general effects of the disease on daily living.
Strong 1993 [23]	Chronic stable plaque psoriasis of an extent sufficient to warrant inpatient admission.	Treated with conventional tar and dithranol during admission ➔ Efamol Marine capsules 500 mg 6# twice daily (containing 80% evening primrose oil and 20% fish oil; 216 mg EPA + 240 mg DHA per day) for up to 7 months after discharge.	Treated with conventional tar and dithranol during admission ➔ placebo capsules 600 mg 6# twice daily (containing liquid paraffin) for up to 7 months after discharge.	Rate of deterioration after discharge (global score, based on BSA, redness, scaling, overall impression, and itch), blood tests.
Veale 1994 [24]	Chronic stable plaque psoriasis and psoriatic arthritis.	12 Efamol Marine capsules daily (total daily dose of 480 mg GLA, 240 mg EPA and 132 mg DHA) for 9 months ➔ placebo capsules for 3 months.	12 placebo capsules daily (containing liquid paraffin) for 12 months.	Chang/improvement in inflammatory joint disease, grip strength, number of active joint, Ritchie articular index, duration of morning stiffness, NASID intake, skin severity (VAS), BSA, itch (VAS), blood tests.

*AE* Adverse event, *ASDAS* Ankylosing Spondylitis Disease Activity Score, *BASDAI* Bath Ankylosing Spondylitis Disease Activity Index, *BASMI* Bath Ankylosing Spondylitis Metrology Index, *BSA* Body surface area involved, *DHA* Docosahexaenoic acid, *DPA* Docosapentaenoic acid, *EPA* Eicosapentaenoic acid, *GLA* Gamma-linolenic acid, *LEI* Leeds Enthesitis Index, *PASI* Psoriasis Area and Severity Index, *PASI 50* at least 50% reduction in the Psoriasis Area and Severity Index score, *SAE* Serious adverse event, *SPARCC* Spondyloarthritis Research Consortium of Canada Enthesitis Index, *VAS* Visual analogue scale

**Fig. 2:**
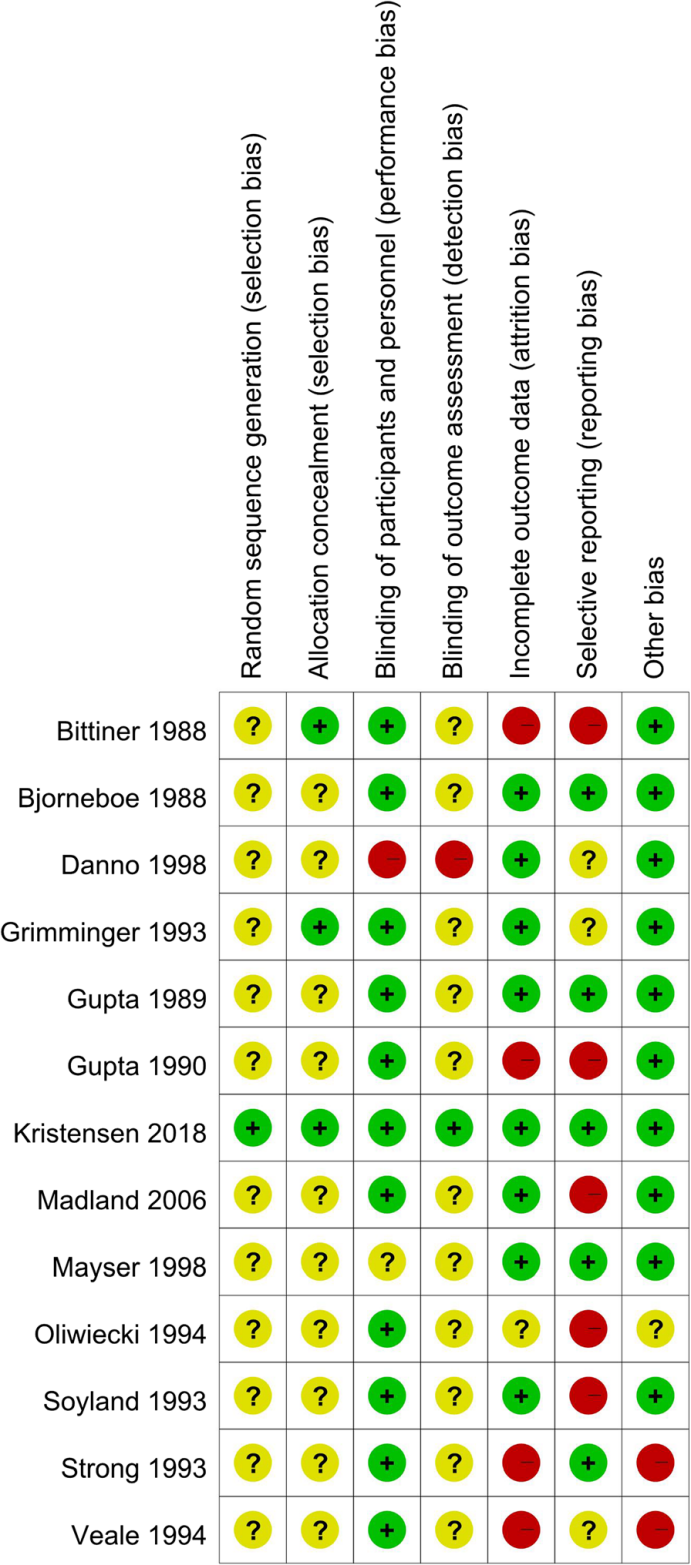
Risk of bias assessment for included studies

The risk of bias assessment of the included RCTs is illustrated in Fig. 2. Eleven out of the 13 included RCTs were published in 1980’s and 1990’s, and thus did not adequately describe the methods of random sequence generation, allocation concealment, and the methods of blinding for outcome assessment [12–17, 20–24]. Hence, these RCTs are rated as having unclear risk of bias on random sequence generation, allocation concealment, and blinding of outcome assessment.

### Effects of fish oil in treating psoriasis

#### Psoriasis area and severity index score

**Fig. 3:**
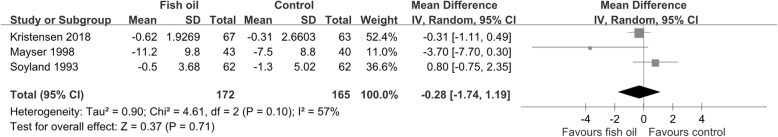
Meta-analysis of efficacy of fish oil supplement in treating psoriasis assessed by Psoriasis Area and Severity Index score

Three RCTs with 337 participants provided data for this outcome [18, 20, 22]. As illustrated in Fig. 3, the meta-analysis found fish oil did not produce significantly greater improvement in the PASI score than the control group (MD − 0.28; 95% confidence interval − 1.74 to 1.19). The statistical heterogeneity across the included RCTs was significant (I^2^ = 57%).

#### Body surface area

Three RCTs contributed data for this outcome [12, 13, 17]. Bittiner et al. [12] reported a trend towards improvement of BSA in the fish oil group without statistical significance after 12 weeks of intervention (treatment group: − 3.0%, *n* = 11; control group: + 0.2%, *n* = 13). Gupta et al. [17] reported significant improvement of BSA in the group of fish oil with concomitant UVB phototherapy than in the control group (treatment group: − 19.0%, *n* = 8; control group: + 11%, *n* = 10; *P* = 0.0001). Bjørneboe et al. [13], however, reported no significant change of involved area during the trial in both the experimental group (*n* = 13) and the control group (*n* = 14).

#### Erythema, scaling, and induration

Six RCTs supplied data for these outcomes [12, 13, 15, 17, 21, 22]. Bittiner et al. [12] reported a significant improvement in the erythema (*P* < 0.05), but not scaling, in the fish oil group (*n* = 11) than in the control group (*n* = 13). Grimminger et al. [15] found the scores of erythema, scaling, and induration in the treatment group (*n* = 9) all improved significantly as compared with those in the control group (*n* = 11) (*P* < 0.05). Gupta et al. [17] also reported significantly better response in respect to the scores of erythema (*P* = 0.02), scaling (*P* = 0.008) and induration (*P* = 0.006) in the treatment group (*n* = 8) than in the control group (*n* = 10). The study conducted by Søyland et al. [22], however, demonstrated no significant differences in the scores of erythema, scaling and induration between the treatment group (*n* = 61) and control group (*n* = 62). Oliwiecki et al. [21] also reported no significant difference in the scores of erythema, scaling and infiltration between the active and placebo-treated groups. Bjørneboe et al. [13] reported no significant change in the scores of erythema, scaling and induration during the trial in both the experimental group (*n* = 13) and the control group (*n* = 14).

#### Pruritus

Two RCTs provided data on pruritus [12, 21]. Bittiner et al. [12] assessed pruritus on a 0 to 5 scale by the participants and found a trend to improvement after 12 weeks’ fish oil supplement when compared to the control group (treatment group: − 1.3, *n* = 11; control group: − 0.3, *n* = 13). Oliwiecki et al. [21] reported no significant difference between the active and placebo groups in the score of pruritus assessed by using a 10-cm linear analogue scale.

#### Adverse events

Nine RCTs provided data concerning this outcome (Table 3) [13–18, 20, 23, 24]. Only a few minor AEs such as mild gastrointestinal adverse effects were reported. In two RCTs where fish oil supplement was given via intravenous route [15, 20], a few participants reported irritations at the injection site. As for the routine laboratory tests, none of the parameters significantly changed during the experimental period [13, 15, 20, 23, 24], except for elevated liver enzyme levels in the study of Danno et al. [14], which could be attributed to concurrent use of etretinate. Overall, there were no significant differences in the incidence of AEs between the fish oil supplement group and control group.

**Table 3:** Adverse events (AEs)

Study ID	Intervention	Adverse events
Bjørneboe 1988 [13]	Fish oil	Routine lab: none of the parameters changed significantly during the trial (CBC, ESR, creatinine, albumin, ALT, GGT, bleeding time, triglyceride, cholesterol).
Danno 1998 [14]	Fish oil + Etretinate	Cheilitis, dry mouth, dry eyes, desquamation of palms, folliculitis, gastric symptoms, and increased liver enzymes. All were mild or tolerable. No significant difference in the incidence between intervention and control groups.
Grimminger 1993 [15]	Fish oil (intravenous)	No obvious side effect except for rare irritations at the site of peripheral intravenous route; routine lab: not change substantially (ESR, CRP, cholesterol, triglyceride, BUN, ALT, GGT, amylase, lipase, coagulation variables).
Gupta 1989 [17]	Fish oil + UVB phototherapy	No side effect was reported in either group.
Gupta 1990 [16]	Fish oil + betamethasone diproprionate cream	An occasional fishy taste upon eructation in one patient on fish oil and one on olive oil therapy; transient diarrhea at beginning of therapy in 2 patients on fish oil
Kristensen 2018 [18]	Fish oil	Mild gastrointestinal adverse effects (9 in intervention group and 6 in control group)
Mayser 1998 [20]	Fish oil (intravenous) + 3% salicyclic acid	Most frequently reported in intervention group: superficial thrombophlebitis; most frequently reported in control group: superficial thrombophlebitis, pruritus, hypertriglyceridemia, and fever; no severe AEs; routine lab: not substantially change (AST, ALT, Alk-P, creatinine, blood glucose, CBC/DC, total cholesterol, triglyceride)
Strong 1993 [23]	Fish oil + evening primrose oil	Blood test: remained unchanged throughout the trial (CBC, urea, creatinine, total lipids, HDL and LDL, cholesterol, triglyceride, electrolytes)
Veale 1994 [24]	Fish oil + evening primrose oil	No significant differences in lab indices including ESR, CRP, hemoglobin, urea, electrolytes, liver enzymes

*Alk-P* Alkaline phosphatase, *ALT* Alanine aminotransferase, *AST* Aspartate aminotransferase, *BUN* Blood urea nitrogen, *CBC* Complete blood count, *CRP* C-reactive protein, *DC* Differential count, *ESR* Erythrocyte sedimentation rate, *GGT* Gamma glutamyl transpeptidase

## Discussion

PASI, which combines the area affected and the severity of the erythema, scaling and induration into a single score, is the most commonly used tool for the measurement of psoriasis severity and treatment response. This study demonstrates that fish oil supplement did not significantly reduce the severity of psoriasis when measured by the PASI score. The effect of fish oil on reducing psoriasis BSA coverage is inconclusive. The studies by Bittiner et al. [12] and Gupta et al. [17] reported benefits but that by Bjørneboe et al. [13] did not. These data could not be pooled due to insufficient data reporting and methodological heterogeneity. The small sample size of these included RCTs is another limitation. When examining the signs and symptoms of psoriasis, the current evidence also shows conflicting results on the improvement of erythema, scaling, induration, and pruritus. Fish oil supplement was found well-tolerated and no severe AEs had been observed.

Five of the included studies did not provide usable outcome data regarding the therapeutic effect for psoriasis, and hence they were not incorporated into our data analysis [14, 16, 19, 23, 24]. Most of them (four out of the five studies) did not support the beneficial effect of fish oil on psoriasis [16, 19, 23, 24]. Danno et al. [14] used a clinical score based on erythema, induration, and scaling of 3 selected plaque lesions to evaluate the severity of psoriasis. They found the number of patients showing excellent improvement (namely, a decrease in the total score of 75% or more) was significantly greater in intervention group than that in control group. Gupta et al. [16] examined the effect of fish oil on maintaining the improvement achieved by topical steroid by comparing the time needed for psoriasis to worsen to pretherapy severity; no significant difference was found between the intervention and control group. Madland et al. [19] reported the severity of psoriasis was unchanged after treatment, but they did not provide data of PASI score. Strong et al. [23] studied the benefit of fish oil on maintaining the improvement obtained by inpatient treatment with conventional tar and dithranol by assessing the rate of deterioration after discharge; no significant difference was noted between the intervention and control group. Veale et al. [24] reported skin disease activity was unchanged in the intervention group but they did not provide data.

Fish oil supplement has been hypothesized to provide beneficial effects on psoriasis through its anti-inflammatory effects. Not surprisingly, there are also many studies focusing on the benefits of fish oil on other diseases with inflammatory properties for example arthritis. One systematic review on the effects of fish oil on arthritis concluded that fish oil might have a small favourable effect on arthralgia, but the evidence was of low quality [25]. Another popular issue is the protective effect of fish oil on cardiovascular disease (CVD) where inflammation plays a central role in its development and complications. Early studies have suggested the benefits of fish oil supplement on CVD. However, a recent meta-analysis involving 10 RCTs with 77,917 participants found no significant benefit of fish oil supplement in fatal or nonfatal coronary heart disease or any major vascular events [26]. Our study adds another piece of evidence that fish oil may have only limited benefit on certain diseases with inflammatory properties.

However, our study results should be interpreted cautiously for the following limitations. Firstly, most of the RCTs were done in 1980s and 1990s, and did not employ a standardized outcome assessment tool to measure the severity of psoriasis for example PASI score. Secondly, these early RCTs did not follow the Consolidated Standards of Reporting Trials (CONSORT) Statement [27] and the reporting of data was inadequate. Therefore, only a limited number of RCTs provided usable data for this study. Thirdly, we excluded non-English studies and thus might have missed relevant data.

There was a previous systematic review of 12 studies on the effects of omega-3 fatty acids on psoriasis [28]. They reported whether the use of omega-3 fatty acids could benefit patients with psoriasis was inconclusive. Our study differed from theirs in the following ways. Firstly, we added three more RCTs including a recent article published in 2018 [18]. Secondly, the previous review included open-label controlled observational studies and studies involving only dietary modification. By contrast, we excluded these low-quality studies and thus provide the best evidence regarding the effects of fish oil supplement in treating psoriasis. Thirdly, no meta-analysis was carried out in the previous review.

## Conclusions

Our study found no consistent evidence supporting the use of fish oil supplement in treating psoriasis. For patients with psoriasis who hope to alleviate their symptoms from dietary changes, we could encourage them to lose weight through dietary control and exercise, for which there is stronger evidence on producing significant improvement in psoriasis severity [29, 30].

## Data Availability

The datasets used and/or analysed during the current study are available from the corresponding author on reasonable request.

## Abbreviations

AE: Adverse event
BSA: Body surface area
CVD: Cardiovascular disease
MD: Mean difference
PASI: Psoriasis Area and Severity Index
RCT: Randomized controlled trial
SMD: Standardized mean difference
